# Predation on endangered species by human-subsidized domestic cats on Tokunoshima Island

**DOI:** 10.1038/s41598-019-52472-3

**Published:** 2019-11-07

**Authors:** Tamao Maeda, Rumiko Nakashita, Kazumi Shionosaki, Fumio Yamada, Yuya Watari

**Affiliations:** 1 Wildlife Research Center, Kyoto University, 2-24 Tanaka-Sekiden-cho, Sakyo, Kyoto 606-8203 Japan; 2 Forestry and Forest Products Research Institute (FFPRI), 1 Matsunosato, Tsukuba, Ibaraki 305-8687 Japan; 3 Amami Wild Animal Research Center, 2662 Ogachi, Tatsugo-cho, Kagoshima 894-0105 Japan; 4 Amami Wildlife Research Center Co., Ltd, 10-11-2F Naze Suehiro-cho, Amami, Kagoshima 894-0027 Japan

**Keywords:** Conservation biology, Invasive species, Stable isotope analysis

## Abstract

It is important to unravel how invasive species impact native ecosystems in order to control them effectively. The presence of abundant exotic prey promotes population growth of invasive predators, thereby enhancing the predation pressure on native prey (hyper-predation). Not only the exotic prey but also feeding by humans is likely to cause “hyper-predation”. However, the contribution of artificial resources to this was underestimated in previous studies. Here, we combined fecal and stable isotope analyses to reveal short- and long-term food habits of free-ranging cats on Tokunoshima Island. Although 20.1% of the feral cat feces contained evidence of forest-living species, stable isotope analysis suggested that the cats were mostly dependent on artificial resources. In addition, a general linear model analysis showed that their diet was strongly correlated with landscape variables. These results indicate that the invasive free-ranging cats are aided by anthropogenic feeding, and they move from the human habituated area to natural areas with high biodiversity. These findings suggest the possibility of human feeding indirectly accelerates the effect of cat predation, and call for a further study on their demography. Cat management mainly involves trapping, but our findings show that educating local residents to stop feeding free-ranging cats and keeping pet cats indoors are also important.

## Introduction

Biological invasion is one of the major causes of biodiversity loss globally^1,2^, especially in insular ecosystems worldwide^3,4^. To effectively control invasive species, it is important to determine how they survive and increase their populations in the areas that they invade. The establishment of populations is one of the essential processes for successful invasion^5,6^; in other words, the impact of the introduced species may not become evident unless they have succeeded in increasing their numbers^7,8^. Various factors, such as species traits, biotic and abiotic environments, and temporal events, can affect a result of such establishment, subsequent increases in abundance, and the ecological impact of an invasive species^8,9^. Hyper-predation is one of the possible processes enhancing the impact of invasive predators^10^. This hypothesis predicts that the presence of abundant primary prey subsidizes the predator population, allowing it to grow and then more severely impact the relatively scarce native prey^11,12^.

Feral cats (*Felis catus*) are among the most influential introduced species^13^, as they have been responsible for the extinction or decrease in numerous mammals, birds, and reptiles, particularly in insular ecosystems^13–19^. Many studies have reported that introduced prey (e.g., European rabbits, black rats, and house mice) are suspected of causing hyper-predation to feral cats on native organisms^11,18,20^. The most well-known example is the case on Macquarie Island, where invasive feral cats caused extinction of the endemic parakeet, *Cyanoramphus novaezelandiae erythrotis*^7^. Feral cats and parakeets coexisted on the island for 60 years, but after rabbits were introduced, feral cats rapidly increased their number and ate up the parakeets in the next 10 years.

Not only the introduced prey but also direct or indirect feeding by humans support cat populations and enhance predation pressure on native species, thereby accelerating their extinction^10,21–23^. Some previous studies reported that cats fed by people can substantially impact on the local ecosystem^21,24^. However, most of the studies were limited to native predators living in urban or peri-urban areas^23^.

**Figure 1:**
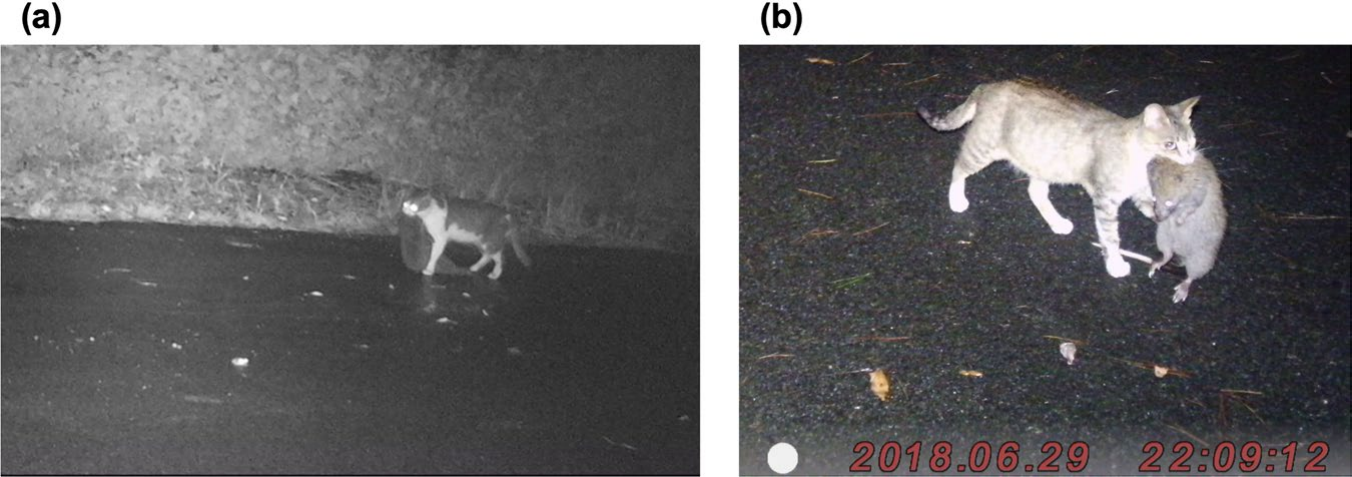
Feral cats killing endemic mammals taken by censor cameras on Tokunoshima Island. (**a**) Amami rabbit, (**b**) Ryukyu long-haired rat. (**a**) Photograph was taken in 2017 and provided with permission by the Naha Nature Conservation Office, Ministry of the Environment. (**b**) Photograph taken in 2018 by the author (Y. Watari, Forestry and Forest Products Research Institute).

Tokunoshima Island is located in southwestern Japan and is a biodiversity hotspot with unique biota that evolved in the absence of native mammalian predators^25^. On this island and adjacent Amami-Oshima and Okinawa Island, free-ranging cats prey on endangered endemic species, such as Amami rabbit (*Pentalagus furnessi*), Ryukyu long-haired rat (*Diplothrix legata*), and spiny rat (*Tokudaia* *tokunoshimensis*, *T. osimensis* and *T. muenninki*)^26,27^, and are believed to be responsible for the population reduction of these species on the islands^28,29^ (Fig. 1). Indeed, a camera-trapping study by the Japanese Ministry of the Environment found a negative correlation between the number of cat appearances and that of endangered Amami rabbits, indicating that the presence of cats can limit the rabbit distribution^30^. The local Tokunoshima Island government and the Ministry of the Environment have been capturing free-ranging cats since 2014 to conserve the endemic species. Cats caught in forests are referred to here as “feral” cats, whereas those caught in residential areas or farmlands are called “stray” cats. “Feral” cats are kept in a shelter, and some can be adopted by new owners after sterilization, whereas “stray” cats are returned to the wild after sterilization (TNR) because it is implicitly assumed that they do not enter the forest and prey on native animals. This management program has been achieving some degree of success, as the monthly route census conducted by the Ministry of the Environment showed that the encounter rates of three endangered mammals, Amami rabbits, Tokunoshima spiny rats (*T. tokunoshimensis*), and Ryukyu long-haired rats, have been increasing while that of cats are decreasing since 2014^31^. It has also been suggested that free-ranging cats are placing substantial predation pressure on native species.

Scientifically, the term “feral” means completely independent and rarely interacting with humans, whereas “stray” cats do not have an owner but still depend on human care^32^. The division of “feral” and “stray” cats by the government suggests their assumption that cats rarely migrate between forests and residential areas, but it is still unclear whether this division is scientifically appropriate due to the lack of studies on free-ranging cats on Tokunoshima Island. Tokunoshima Island is characterized by small forested areas^33^, so it is rather likely that “feral” cats and “stray” cats have access to both wild animals in the forest and artificial food in the villages. Even the core area of the forest is only a few kilometers away from farmlands, which is close enough for free-ranging cats to access both^34–37^. Human garbage was found in 7.1–50% of cat feces on the northern part of Okinawa Island (Yambaru) and Amami-Oshima Islands^26,27^. However, fecal analysis cannot be used for accurate estimation of the actual dependence on artificial resources, owing to the different digestibility among food items^38^. Instead, stable isotope analysis is a powerful method to clarify the dependence of subjects on a given food item^39^. If the so-called “feral” cats are fed with human food, in order to reduce the high predation on native species, it could be effective to keep domestic cats indoors or stop feeding cats without owners. Evidence of resource dependence of cats would provide strong support in promoting public awareness of this issue and in developing an effective strategy for conserving native species.

This study evaluated the diet of free-ranging cats on Tokunoshima Island to verify our hypothesis that both “feral” and “stray” cats are accessible to the forest and the residential area and they are highly dependent on the human-provided resources, although they also predate endemic species in the forest. This dietary state of cats is one of the necessary conditions for the occurrence of hyper-predation induced by human-derived food resources. We specifically addressed three questions: (1) Do “feral” cats eat endangered species more often than “stray” cats? (2) Does providing artificial food substantially support diet of free-ranging cats? (3) Do the contributions of forest prey (including endangered species) and artificial food of free-ranging cats differ among capture locations with different surrounding landscapes? To answer these questions, we collected fecal and hair samples from trapped free-ranging cats and conducted fecal and stable isotope analyses.

## Results

### Fecal analysis

**Figure 2:**
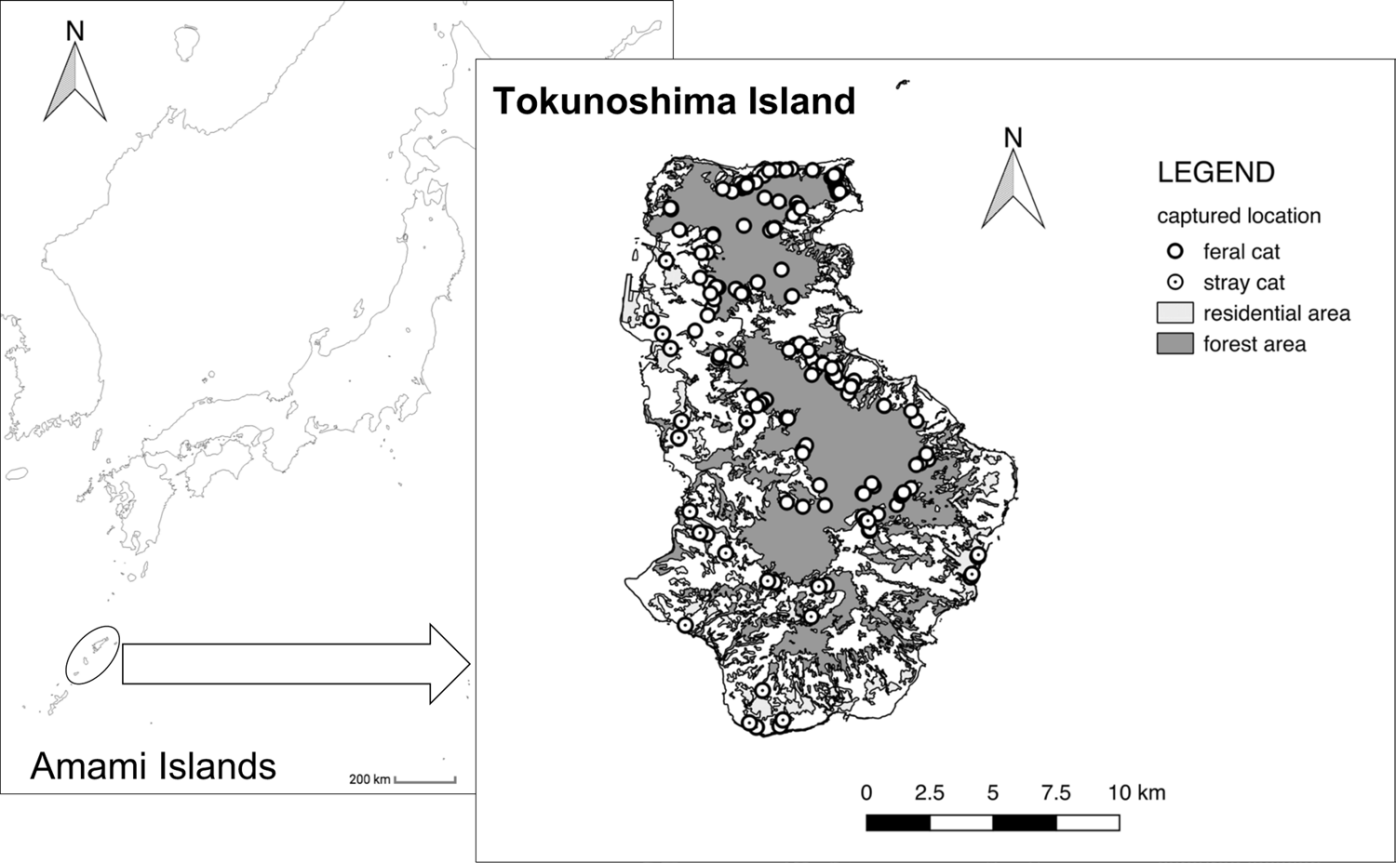
Map of Tokunoshima Island, which is located on the Amami Islands in the Rukyu Chain and the capture location of cats.

In total, 208 “feral” cats (75 females, 123 males, and 10 unidentified) and 54 “stray” cats (22 females, 30 males, and 2 unidentified) were captured, and 198 fecal samples (from 174 “feral” cats and 24 “stray” cats) were obtained (Fig. 2). A total of 13.4% of the cats (35; 31 “feral” and 4 “stray”) were ear-tipped, which means that they had been captured as “stray” cats and sterilized (Table S1). Evidence of forest-living species and farmland-living animals were found in at least 17.7% and 30.8% of the fecal samples, respectively (Table 1). Overall, 23.7% of fecal samples contained artificial objects, such as plastic or paper (Table 1). In addition, 20.1% of the fecal samples from “feral” cats contained evidence of forest animals, which was significantly higher than the rate for “stray” cats (0.0%) (Fisher’s exact test, p < 0.01). No significant difference was observed in the occurrence frequency of farmland species (“feral”: 31.6%, “stray”: 20.8%) and artificial objects (“feral”: 24.1%, “stray”: 20.8%) (p > 0.05). Six threatened species (at least 43 individuals) were detected in 13.5% of the fecal samples: Ryukyu robins (*Erithacus komadori komadori*), which are endemic to southern Japan^40^, and Amami rabbit, Ryukyu long-haired rat, Tokunoshima spiny rat, *Crocidura* spp., and Amami tip-nosed frog (*Odorrana amamiensis*), which are endemic to the Ryukyu Islands^41^. Black rats and chickens were the only non-native prey in this study^42^.

**Table 1:** Result of fecal analysis. FO, frequency of occurrence of items and NI, number of prey items.

Items	FO (%)			NI			Contribution to DCB (%)	body weight (g)	IUCN Red List 2017	Reference for body weight
	TOTAL (n = 198)	feral (n = 174)	stray (n = 24)	TOTAL (n = 198)	feral (n = 174)	stray (n = 24)				
**Forest species**	**17**.**7**	**20**.**1**	**0**.**0**	**40**	**40**	**0**	**15**.**5**			
*Diplothrix legata*	6.1	6.9	0.0	12	12	0	7.7	483	EN	^27^
*Pentalagus furnessi*	4.0	4.6	0.0	8	8	0	6.7	2880*	EN	^75^
*Tokudaia tokunoshimensis*	3.0	3.4	0.0	6	6	0	1.3	162.4	EN	Yamada (unpublished)
*Erithacus komadori komadori*	0.5	0.6	0.0	1	1	0	0.0	22.4	VU	^55^
*Turdus pallidus*	0.5	0.6	0.0	1	1	0	0.1	78		^27^
*Odorrana amamiensis*	0.5	0.6	0.0	1	1	0	0.1	60	VU	^76^
*Diestrammena gigas*	5.1	5.7	0.0	10	10	0	0.0	3		^76^
*Thereuopoda clunifera*	0.5	0.6	0.0	1	1	0	0.0	3.5		^27^
**Farmland species**	**30**.**8**	**31**.**6**	**20**.**8**	**70**	**65**	**5**	**8**.**7**			
*Rattus rattus*	24.2	26.4	20.8	53	48	5	6.9	98		**
Crocidura spp.	6.6	7.5	0.0	15	15	0	0.1	7	(C. orii) EN (C. watase) NT	^77^
*Gallus gallus domesticus*	1.0	1.1	0.0	2	2	0	1.7	1500-*		^78^
**Unknown**	—			—			—			
*Horornis diphone*	1.0	1.1	0.0	2	2	0	0.0	15.8		^79^
Unidentified birds	15.7	17.8	0.0	31	31	0	—	—	—	
Amphibians/Reptiles	3.5	4.0	0.0	7	7	0	—	—	—	
Orthoptera	8.6	6.3	25.0	17	11	6	—	—	—	
Mantodea	1.0	1.1	0.0	2	2	0	—	—	—	
Coleoptera	3.0	2.9	4.2	6	5	1	—	—	—	
Unidentified insects	41.4	46.6	4.2	82	81	1	—	—	—	
Crustacea	1.5	1.7	0.0	3	3	0	—	—	—	
Gastropods	0.5	0.0	4.2	1	0	1	—	—	—	
**Artificial objects**	**23**.**7**	**24**.**1**	**20**.**8**	**47**	**42**	**5**	—	—	—	
**Plants**	**42**.**4**	**32**.**2**	**50**.**0**	**84**	**72**	**12**	—	—	—	

*As these species (*Pentalagus furnessi* and *Gallus domesticus*) were heavier than the max DCB, we used max DCB to calculate the contribution to DCB.

**We calculated the mean body weight of the captured *Rattus rattus* (n = 12).

The average weight of captured cats was 3.3 ± 1.0 kg (range: 1.0–6.0 kg; male: 3.6 ± 0.9 kg, female: 2.7 ± 0.7 kg). Thus, the estimated average daily consumed biomass (DCB) of cats was 379 ± 143 g (range: 146–629 g). The body mass of Amami rabbits and chickens exceeded the maximum DCB of the captured cats, so for these species, we used the maximum DCB (629 g) as the weight. The results showed that this method explained only 24.2% of the cats’ diet (forest animals: 15.5%, farmland animals: 8.7%). The contribution from forest animals was mostly based on two endangered mammals, Ryukyu long-haired rats (7.7%) and Amami rabbits (6.7%), and that of farmland animals on black rats (6.9%).

### Isotopic mixing model

**Figure 3:**
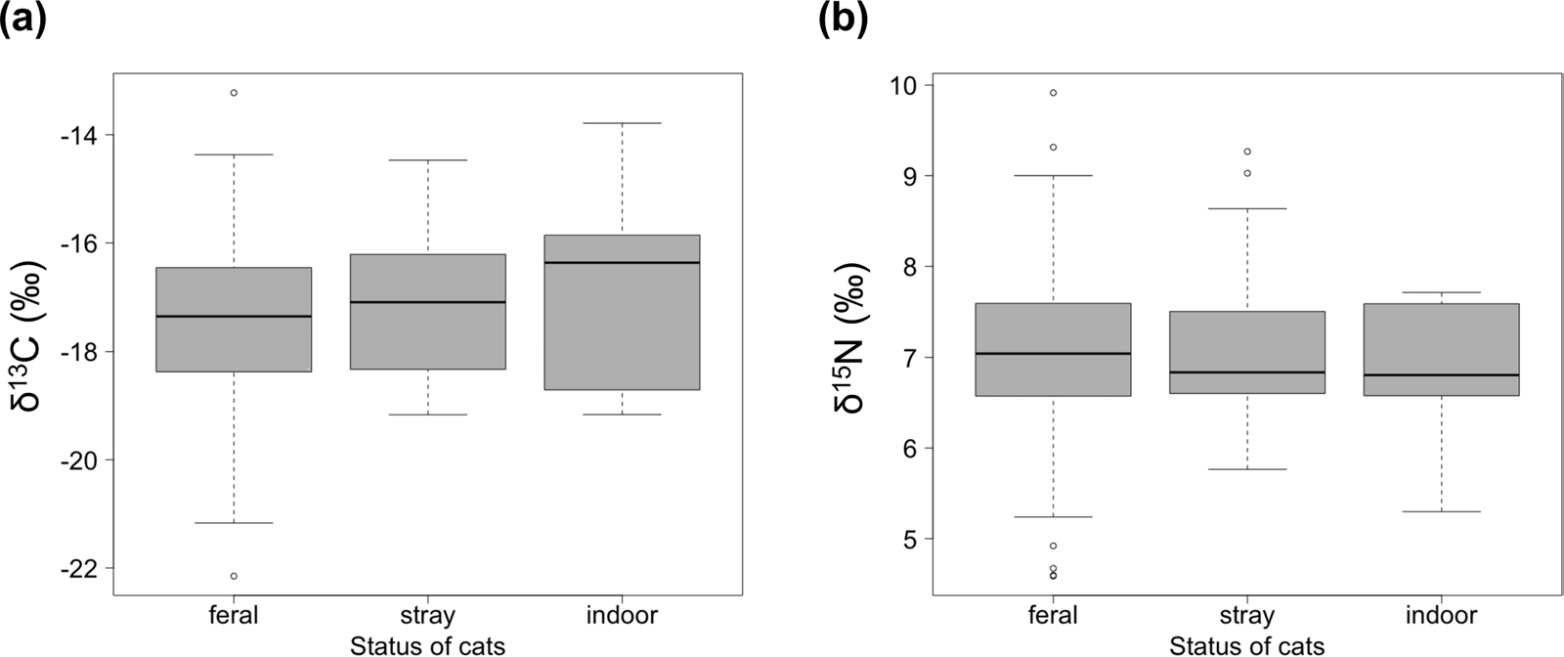
Stable isotope ratios of (**a**) carbon and (**b**) nitorogen in feral, stray, and indoor cats. Error bars represent standard deviations and the bold bars in the box represent medians.

**Figure 4:**
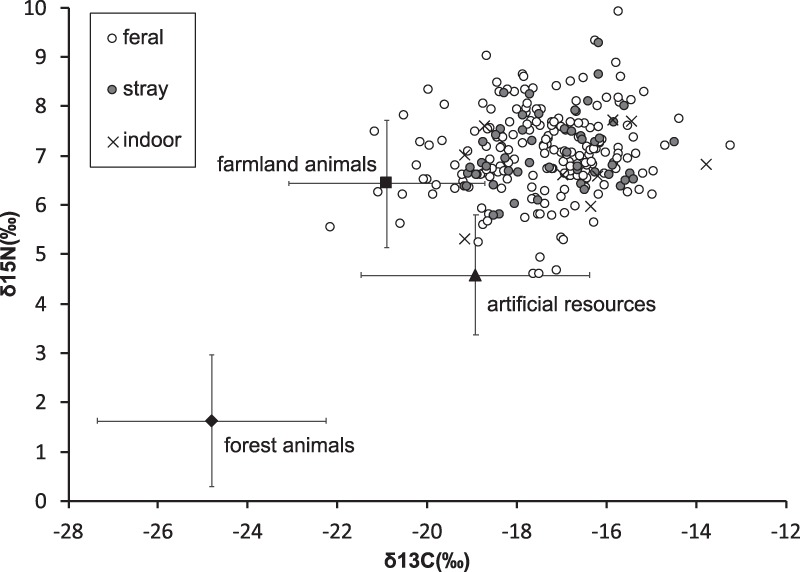
Stable isotope ratios of the cats and their potential resources. Error bars represent standard deviations. Farmland animals and artificial resources are represented by black rats and pet food, respectively. The stable isotope ratio of the forest animals was the average of Amami rabbits and Ryukyu long-haired rats.

We analyzed the hair of 189 “feral” cats, 52 “stray” cats, and 9 indoor cats. The stable isotope ratios of carbon were −17.4 ± 1.4‰, −17.2 ± 1.2‰, and −16.9 ± 1.7‰, and those of nitrogen were 7.0 ± 0.9‰, 7.1 ± 0.8‰, and 6.8 ± 0.8‰, respectively (Figs 3 and 4). Analysis of variance (ANOVA) detected no significant difference in the stable isotope ratio among “feral”, “stray”, and indoor cats [δ^13^C: F(2,247) = 1.15, p = 0.319; δ^15^N: F(2,247) = 0.43, p = 0.651].

We obtained different brands of dried cat food (n = 9) and hair samples of Amami rabbit (n = 7), Ryukyu rat (n = 7), and black rats (n = 7) as candidate cat dietary resources. The stable isotope ratios of carbon in forest animals, farmland animals, and artificial resources were −24.8 ± 2.6‰, −20.9 ± 2.1‰, and −18.9 ± 2.5‰, and those of nitrogen were 1.6 ± 1.3‰, 6.4 ± 1.3‰, and 4.6 ± 1.2‰, respectively. ANOVA on the isotope ratio revealed significant variation among resources, δ^13^C: F(2,33) = 16.43, p < 0.001; δ^15^N: F(2,33) = 43.84, p < 0.001. Post hoc Tukey’s test showed that forest animals had significantly lower δ^13^C than the rest (p < 0.01). Farmland animals had the highest δ^15^N, artificial resources had the second highest, and forest animals had the lowest (p < 0.01).

The changes in δ^13^C and δ^15^N of sheltered cats versus time are shown in Fig. S1. The estimated asymptotes of the regression model (TEF) of δ^13^C and δ^15^N were 2.3 ± 0.3 and 2.8 ± 0.1, respectively.

**Figure 5:**
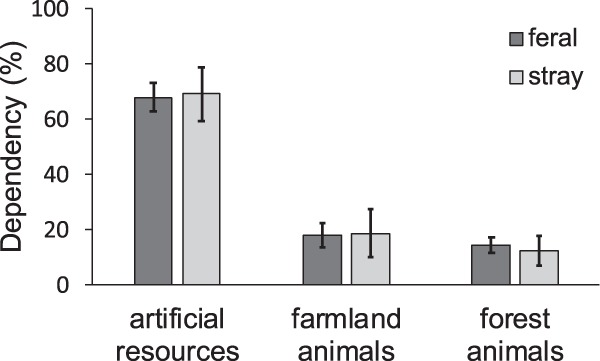
Dependency of captured cats on three resource types, including artificial resources, farmland animals, and forest animals. Error bars represent the 95% high density region. Values were derived from stable isotope analysis in R (SIAR)^80^.

The stable isotope analysis in R (SIAR) indicated that artificial resources were the largest component in energy consumption of the “feral” (67.8%; 95% highest density region: 62.8–72.8%) and “stray” cats (69.0%; 59.3–78.8%), followed by farmland animals (“feral”: 17.9%; 13.4–22.3%, “stray”: 18.5%; 9.7–27.3%) and forest animals (“feral”: 14.3%; 11.6–17.1%, “stray”: 12.4%; 9.7–27.3%) (Fig. 5). In addition, this high dependence on artificial resources remained when we conducted the SIAR only on cats that had evidence of forest animals in their feces (Fig. S3), even though they tended to be captured closer to the forest than the undetected individuals (Fig. S2, Appendix). The estimated dependence on wild animals is generally consistent with the contribution calculated from the fecal analysis (forest animals: 15.5%, farmland animals: 8.7%).

### Effect of landscape elements on the cat diet

In factor analysis, residential area coverage and building density both loaded positively on factor 1, whereas forest coverage and farmland coverage loaded negatively and positively on factor 2, respectively (Table S2). The results of the general linear model (GLM) revealed that dependence on artificial resources was positively correlated with residential area coverage (factor 1) and weight, dependence on farmland animals was positively correlated with farmland coverage (factor 2), and dependence on residential area was positively correlated with forest coverage (factor 2) but negatively correlated with body weight (Table 2).

**Table 2:** The results of model averaging of the selected general linear model (GLM) with corrected version of Akaike information criterion Δ (AIC) < 2 (family = Gaussian). The heading row represents the response variables and dependency on each of the three resources; *p < 0.05, **p < 0.01, ***p < 0.001.

Explanatory variables	Artificial Resources			Farmland Animals			Forest Animals		
	Estimate (×10^−3^)	z	p	Estimate (×10^−3^)	z	p	Estimate (×10^−3^)	z	p
Residential Area (Factor1)	10.65	2.59	**	−0.11	0.06		−5.83	0.83	
+Farmland/−Forest (Factor2)	−0.10	0.08		16.08	2.84	**	−2.09	2.85	**
weight	16.62	3.54	***	0.72	0.27		−22.15	3.33	***
sex (male)	−4.43	0.54		1.14	0.23		0.56	0.13	
MEM1	−9.43	2.24	*	−11.83	2.29	*	22.82	3.53	***
MEM2	−0.38	0.21		11.16	2.13	*	−6.36	0.89	
MEM3	0.00	0.00		−10.79	2.09	*	5.85	0.84	
MEM4	−0.03	0.03		0.02	0.01		−0.03	0.02	
MEM5	−1.31	0.45		−16.08	3.24	**	24.45	3.93	***
MEM6	−0.04	0.04		14.00	2.62	**	−14.62	2.18	*

## Discussion

Our study revealed the dietary habits of invasive cats on Tokunoshima Island by combining fecal analysis with stable isotope analysis. A dietary difference between “feral” and “stray” cats was only detected in the fecal analysis, which reflects the diet of the past few days in each habitat where the cats were captured. According to the isotopic mixing model, the cats’ long-term diet did not differ significantly, and even the cats that had evidence of forest animals in their feces largely depended on artificial resources. It is likely that the cats visited the forests for a few days, where they hunted native endangered animals, and then traveled back to the villages to eat cat food, which was their main source of food. In addition, many of the captured “feral” cats were ear-tipped, i.e. they had been captured as “stray” cats in the residential area, and this ratio was not significantly different from that of “stray” cats. It also suggests movements of cats between the forest and the villages. The endemic mammal population has been substantially impacted by cats^28,31^, whereas our study shows that cats themselves depend on human-derived resources. In addition, both stable isotope analysis and fecal analysis suggested relatively low dependence on farmland animals, namely, black rats, unlike the case on many other islands where introduced prey is available^16^. This may be due to the low density of black rats in the forests of Tokunoshima Island^30^. In fact, a rat population survey by Jogahara showed that spiny rats dominated in the forests, whereas black rats were rarely captured (unpublished data).

Free-ranging unowned cats are usually divided into two categories: feral cats, which depend on native resources, and stray cats, which depend on artificial resources^32^. The GLM results show that dependence on each resource had a positive association with the land use where it was assumed to have been obtained. The dependence on forest-living animals increased at capture locations close to the forest, suggesting that feral and stray cats cannot be clearly separated and that free-ranging cats can be a threat to native species, particularly in human residential areas adjacent to the natural environment. If the forest was large enough, their dependence on artificial resources would be minimized, which would mean that pure feral cats would breed. In other words, small habitats, such as the forest on Tokunoshima Island, may be more susceptible to the effect of human-derived resource subsidization as well as other kinds of effects^27^. In addition, roads may also increase the accessibility of forests from residential areas, as carnivores often prefer to move on tracks or roads^43,44^, and people can use them to abandon their pets in natural areas more easily.

According to the local Tokunoshima Island government, 2,797 “stray” cats were captured and sterilized from April 2014 to March 2018. However, only 13% of the captured cats were ear-tipped, and this proportion is not increasing. The results imply the huge number of cats on the island and their successful reproduction. We assume that stable and inexhaustible human-derived resources enable cats to sustain this large population, but further investigation would be needed to evaluate the effect of artificial resources on cat demographics. Although the management may have been successful in achieving some recovery of endemic mammals, it might be difficult to eliminate free-ranging cats unless the resource subsidization by humans is controlled.

Overall, our study indicates that invasive free-ranging cats depend on anthropogenic feeding, the effect of which may reach far from the habituated area to natural areas with high biodiversity. This finding provides new insight into how to best manage invasive cat populations. The main predator management options are trapping, which has occurred on Tokunoshima Island, and lethal control^10,45^. However, owing to the necessity for continuous intervention, such management is often very expensive, which sometime leads to failure of the whole project^45^. In the case of human-driven hyper-predation, preventing the access of cats to artificial resources is a more cost-effective way of reducing the predator population in the long term. Studies on this topic will be important to better plan predator control programs. If the local people provide additional resources to predators without being aware of their impact on the ecosystem, introducing scientific evidence for human-driven hyper-predation may improve their awareness of how to treat their pets and neighboring wildlife appropriately. Although this method would effectively reduce the predator population in the long term, a sudden decrease of resource subsidization usually causes a temporary increase in predation pressure on the native prey^29,46^. Several methods should thus be combined, including lethal control and resource subsidization control, to develop an effective conservation strategy.

Tokunoshima Island has regulations about keeping pet cats indoors and prohibits the feeding of unowned cats. However, as the mixing model showed high dependence on artificial resources, it is likely that many people are not following these regulations. Our study provides important scientific evidence to prove the need to educate people and support such regulations. In addition, this study suggests the potential impact of TNR cats on endangered species in the short term. The effectiveness and validity of this method should thus be reconsidered.

Our study also points out the limitations of fecal analysis in detecting the effect of anthropogenic subsidization because only 24% of the feces contained artificial objects, despite the high dependence suggested by the isotopic mixing model. Previous studies detected artificial materials in cat feces or stomach/gut contents, but they were usually excluded from the subsequent dependence calculation due to the low frequency of occurrence of artificial items and the difficulty in estimating their mass^27,47,48^. It is possible that these studies largely underestimated the effect of feeding by humans; thus, it would be better to combine multiple methods, such as stable isotope analysis, to obtain accurate estimates of the diet^49^.

Although our study suggested the occurrence of resource subsidization by humans on the cat population, we still lack the demographic and ethological studies on free-ranging cats and endemic species. The high dependency of diet does not necessarily mean that the resource is essential for supporting the population because it might be just a consequences of resource selectivity. It is required to research whether human feeding actually gives positive impacts on cat population, and predation by cats gives negative impacts on the endemic mammals in order to fully verify our assumption of hyper-predation by human feeding and to understand its precise process and consequences. For example, if cat population increases due to anthropogenic resource subsidization, the density of cats may positively correlate with that of residents.

In conclusion, our study provides strong circumstantial evidence of anthropogenic resource subsidization on free-ranging cats. It points out the possibility of human-driven hyper-predation and provides important support for promoting local and global invasive predator control management.

## Methods

### Study area

Tokunoshima Island (N 27°45′, E 128°58′) is located in the Ryukyu Archipelago, southwestern Japan (Fig. 2). It has an area of 247.85 km^2^ and a population of ~25,000 inhabitants^33^. Tokunoshima Island is in a subtropical region with high precipitation (mean temperature, 21.6 °C; mean annual rainfall, 1912 mm). It has mountains (highest peak, 645 m) that run north to south, surrounded by a plateau of Ryukyu limestone. This island consists primarily of crop fields and broad-leaved evergreen forests, covering 28% and 43% of the area, respectively. Sugarcane predominates as a crop field, and evergreen oak species, such as *Castanopsis sieboldii* and *Quercus miyagii*, are the major components of the forest.

The Ryukyu Archipelago, especially the Central Ryukyus, including Tokunoshima Island, had separated from the Eurasian continent at least by the late Miocene (11.63–5.33 million years ago)^50^. The native top predators are habu vipers (*Protobothrops flavoviridis* and *Ovophis okinavensis*)^51^, and a great number of endemic species and subspecies have evolved in the absence of native mammalian predators^40^. Many of them are highly endemic, e.g., Amami rabbit is endemic to Tokunoshima and adjacent Amami-Oshima Island; Ryukyu long-haired rat is endemic to Tokunoshima, Amami-Oshima, and the northern part of Okinawa Island; and Tokunoshima spiny rat only lives on Tokunoshima Island^41^. Most of the endemic species, including these three mammals, are threatened and listed on the Red List of the International Union for Conservation of Nature and Natural Resources (IUCN 2017) and the Japanese Ministry of the Environment (2017).

### Sample collection and dietary analysis

Cat diets are usually evaluated by stomach content or fecal analysis^16,52^. These methods provide a short-term picture of the diet but underestimate the contribution of highly digestible material, such as pet food, and immeasurable objects, such as garbage. Stable isotope analysis is an alternative way to identify major food items, as it provides information on the long-term contributions of major foods and is less affected by differences in digestibility. However, as potential prey species may have similar isotopic values, taxonomic resolution is occasionally low, particularly for generalist and opportunistic predators such as cats. A combination of fecal and stable isotope analyses can compensate for the other’s disadvantages and reveal a more precise, long-term dietary history^53,54^.

In this study, hair and fecal samples were obtained from feral and stray cats captured in the population control program conducted on Tokunoshima Island. Feral cats were trapped in forest areas more than 500 m away from villages, whereas stray cats were trapped in or around villages. Both feral and stray cats were captured using metal box traps with cat food or fried chicken inside and then brought to an animal hospital for sterilization. Hair samples were collected by a vet during surgery. After sterilization, the cats were kept in separate cages for a few days for the collection of feces. Feral cat samples were collected from December 2014 to January 2018, and stray cat samples were collected in November 2017. The capture location, capture date, sex, and body weight were recorded for each cat. When ear-tipped cats, individuals which had experienced TNR in the past, were captured after November 2017, we compared them with the photos of stray cats captured previously to make sure that they had not yet been sampled.

### Fecal analysis

Fecal samples were kept in plastic bags frozen at −20 °C. The feces were washed over a 1-mm mesh sieve under a stream of water and dried in an oven at 65 °C for more than 12 h. Each food item was identified to the species level and assigned to one of the four main habitat types: forest-living species (forest animals), farmland- and residential area-living species (farmland animals), artificial resources, and unidentified animal/plant materials. Most of the species exclusively live in either forest or non-forest areas^30,41,55^, except *Horornis diphone*; thus, we categorized this species as “unidentified.” We categorized black rats as “farmland animals” because black rats rarely occurred in the forest where endangered species inhabited. Unidentified animal/plant materials were excluded from the following dietary analysis. The number of individual prey in each scat was counted based on distinctive bones, such as jaws and incisors. We estimated the frequency of occurrence and the minimum number of individuals for each prey species.

To narrow down the candidate prey species for the stable isotope analysis, we estimated the contribution of each prey species to DCB of cats following the methods of Bonnaud *et al*.^56^ and Shionosaki *et al*.^27^. As cats usually defecate once per day^57,58^, the formula can be written as follows:

1 $${\rm{Contribution}}=\frac{{\rm{mean}}\,{\rm{body}}\,{\rm{weight}}\,{\rm{of}}\,{\rm{prey}}\times N{\rm{I}}/n}{{\rm{mean}}\,{\rm{DCB}}\,{\rm{of}}\,{\rm{cats}}}\times 100\,( \% )$$

where *n* is the total number of scat samples and NI is the minimum total of individual prey found in the scats. DCB of free-living, eutherian predators can be estimated using the allometric equation: DCB = 3.358 × (*body weight of predator*)^0.813^ × 2.86/18 (g)^59,60^. Here, 2.86 is included to account for the 65% water content of prey (100/(100 − 65) = 2.86), and 18 represents the mean energy content in kJ of metabolizable energy per gram of dry prey^59,60^. We set the upper limitation of body weight of prey as the maximum DCB of the cats because, when cats catch large prey, they are likely to eat some and leave the rest^61^. We previously ran SIAR with prey species whose contribution was >1% and obtained the result which reveals that the smallest 95% HDR interval was 3.0%. Thus, we defined important prey species for cats as those whose contribution was >3%. The results showed that two forest-living species (Amami rabbits and Ryukyu long-haired rats) and one farmland- and residential area-living species (black rats) satisfied this threshold and were used for the following stable isotope analysis. We compared the frequency of occurrence of each prey category between feral cat feces and stray cat feces using Fisher’s exact test.

### Stable isotope analysis

The entire hair of cats and prey species, including the root, was plucked and kept in a plastic bag. Hair samples of indoor pet cats, which had been fed only pet food, were also collected for comparison with the feral and stray cats. In addition, hairs of “sheltered cats” (cats captured as feral and thereafter kept in a shelter for 23–536 days and supplied with pet food) were taken to estimate the trophic enrichment factor (TEF). All hair samples were provided by the cat population control programs by the local government on Tokunoshima Island and the Japanese Ministry of the Environment, which were carried out in accordance with the Act on Welfare and Management of Animals and Protection and Control of Wild Birds and Mammals and Hunting Management Law, respectively.

We assumed three types of dietary resources for the cats according to the results of the fecal analysis: forest animals (Amami rabbit and Ryukyu rats), farmland animals (black rats), and artificial resources (pet food). Black rats were captured using metal box traps in villages and farmlands, and hairs were plucked from their necks. Samples of endangered Amami rabbits and Ryukyu rats were obtained, with permission, from frozen carcasses (mostly killed in traffic accidents) stored by the Ministry of the Environment. As a representative artificial resource, pet food was analyzed because it is the major food people feed to cats on Tokunoshima Island, and it is likely to have an isotope ratio similar to that of other possible artificial resources, such as leftover meals and garbage, as the ingredients of pet food resemble the human diet, namely, grains, fish, meat, and soy^62^.

We analyzed the carbon and nitrogen stable isotope ratios in the samples using a method similar to that of Mizukami *et al*. (2005a, 2005b)^63,64^. The hair was rinsed with a 2:1 chloroform–methanol solution to remove lipids and was air-dried. It is recommended that lipids be removed because they are depleted in ^13^C relative to carbohydrates and proteins^65^, and their amount can vary greatly among individuals^66^. Pet food was dried in an oven at 65 °C for >12 h and pulverized with a food mill. Samples were enclosed in a tin cup and combusted in a FlashEA 1112 elemental analyzer (Thermo Fisher Scientific, Bremen, Germany) interfaced to a Delta V isotope ratio mass spectrometer (Thermo Fisher Scientific). The analytical errors for the isotope analysis were within 0.1‰ for *δ*^13^*C* and 0.2‰ for *δ*^15^*N*.

### Isotopic mixing model

The Bayesian mixing model SIAR was applied using the R package “siar” to estimate the cats’ dependence on each resource^67^. The SIAR model is suitable for Markov chain Monte Carlo (MCMC) methods in finding a plausible dietary composition using Dirichlet prior distribution^67^.

It is usually assumed that δ^13^C increases by 0‰–1‰ and δ^15^N by 3.4‰ from one trophic level to the next^65,68^. However, TEFs can differ among environments, trophic levels, tissues, species, and sample treatment procedures^39,53,54,69,70^. To estimate the appropriate TEF for this study, we analyzed the isotope ratio of sheltered cats. As sheltered cats were fed the same pet food after being captured, their isotope ratio would converge with the isotope ratio of pet food +TEF. We used the asymptotic exponential model *y* = *Ae*^*Bx*^ + *C* with ΔδX (δX of a shelter cat – δX of the pet food; X = ^13^C or ^15^N) as a response variable and time in days that a cat spent in the shelter as an explanatory variable. We defined TEF as the estimated asymptote (parameter C).

We calculated the isotope ratio of forest animals (Amami rabbits and Ryukyu long-haired rats), farmland animals (black rats), and artificial resources (pet food). The isotope ratio of forest animals was defined as the mean isotope ratio of the two species.

The MCMC was run 50,000 times, discarding the first 5,000 samples and thinning by 10 {i.e., [number of groups × (number of sources + number of isotopes)] = 2 × (3 + 2)} to avoid sample autocorrelation.

### General linear model

We used a GLM with a Gaussian structure (link = “identity”) under the R environment to analyze the effect of landscape elements on the diet of free-ranging cats. The response variable was the dependence of an individual cat on each of the three resources estimated by stable isotope analysis, which was taken as the arcsine square root transformation. The explanatory variables were land-use variables surrounding capture locations, sex, body weight, and spatial autocorrelations.

Land-use variables included forest, residential area, and farmland coverage and density of buildings. The land coverage data were obtained from the National Land Numerical Information download service, and building locations were taken from the Geospatial Information Authority of Japan website. Cats fed by humans usually do not travel more than 300–800 m from the feeder’s house^71,72^. In addition, feral cats in our study were defined as those captured at least 500 m away from villages. Thus, we created radius buffers of 100, 200, and 500 m from the capture location. As land-use variables were strongly correlated with each other, we summarized them using an explanatory factor analysis. An exploratory factor analysis was conducted using maximum likelihood factor extraction to determine the factor structure of the landscape elements around the 165 capture locations of the 237 cats. The reason for using factor analysis rather than a principal component analysis is that the axes of a factor analysis are easier to interpret in terms of land-use patterns. A parallel analysis recommended a two-factor solution. We employed Promax (oblique) rotation to interpret the two factors.

Spatial autocorrelation variables were added to consider the effect of spatial proximity on the cat diets. We constructed Moran’s eigenvector maps (MEM) using the Delaunay triangulation method and calculated the scores for each capture location using the R package “adespatial”^73^. As the larger MEM values representing a finer spatial structure may overlap with land use within the 100–500 m radius buffer, we considered MEM1-10 first and then selected the model. The largest significant MEM value was MEM6, so we used only MEM1–6 in subsequent analyses.

The model was selected using a multi-model inference approach. We used “MuMIn” package^74^ to produce all subsets of models based on the global model and ranked them based on the corrected version of Akaike information criterion (AIC). We used model averaging to produce the averaged parameter estimates of all models with ΔAIC < 2.

## Supplementary information

Supplementary Appendix
