# Supplementary material for: Predation on endangered species by human-subsidized domestic cats on Tokunoshima Island
Source: Sci Rep. 2019 Nov 7;9:16200. doi: 10.1038/s41598-019-52472-3 (PMC6838317; doi:10.1038/s41598-019-52472-3)

Predation on endangered species by human-subsidized domestic cats on Tokunoshima Island

Tamao Maeda[1], Rumiko Nakashita[2], Kazumi Shionosaki[3, 4], Fumio Yamada[2], and Yuya Watari[2, *]

[1]Wildlife Research Center, Kyoto University, 2-24 Tanaka-Sekiden-cho, Sakyo, Kyoto, 606-8203, Japan
[2]Forestry and Forest Products Research Institute (FFPRI), 1 Matsunosato, Tsukuba, Ibaraki 305-8687, Japan
[3]Amami Wild Animal Research Center, 2662 Ogachi, Tatsugo-cho, Kagoshima 894-0105, Japan
[4]Amami Wildlife Research Center Co., Ltd, 10-11-2F Naze Suehiro-cho, Amami, Kagoshima 894-0027, Japan
*ywatari@affrc.go.jp

Supplementary Appendix

We compared the cats' isotope ratio and the land use variables of their capture locations based on the availability of forest-living animals in their feces to ensure our assumption that free-ranging cats mainly feed on artificial resources and on native animals as an additional resource.

Among the 262 captured cats, 180 (forest animal detected: n = 33, undetected; n = 147) had both results of the fecal and stable isotope analyses. The detected individuals for the land use variables analysis had significantly lower scores for factor 1 (Welch two-sample test; $t = -3.43$, $df = 156.2$, $p < 0.01$) and factor 2 ($t = -4.70$, $df = 57.7$, $p < 0.01$) (Fig. S2). As factor 1 was labeled the "residential area" indicator and factor 2 was "+farmland/-forest" (Fig. S1), this result suggests that the cats were captured closer to the residential area, and farther from the forest.

The average $\delta^{13}C$ values of detected and undetected individuals were -17.3 ± 1.3 and -17.4 ± 1.4, and those of $\delta^{15}N$ were 7.1 ± 0.7 and 7.0 ± 0.9. Welch's two-sample test found no significant difference between them ($\delta^{13}C$: $t = 0.50$, $df = 48.9$, $p = 0.63$, $\delta^{15}N$: $t = 0.54$, $df = 62.9$, $p = 0.59$), which means they have similar long-term dietary habits. In addition, the stable isotope mixing model in R (SIAR) result also suggested that the detected and undetected individuals had high dependency on artificial resources (Fig. S3).

Table S1. Number of captured cats in each year and the ratio of ear-tipped cats. Many of the "feral" cats were ear-tipped, i.e. they were once captured as "stray" cats in the villages. According to the Pearson's chi-squared test with Yates' continuity correction, the ratio of ear-tipped cats did not differ significantly between "stray" and "feral" cats (2017: χ^2= 0.47, p= 0.49 ; 2018 from February: χ^2= 0.89, p=0.35). In addition, the ratio of ear-tipped cats has not been increasing in spite of the continuous effort of TNR. *Stray cats were sampled during November in 2017. ** This data was provided by K. Kazato. "Feral" and "stray" cats were captured during February to October and April to November, 2018, respectively. We did not used these cats as samples in this study.

year	**feral**	ear-tipped	**stray**	ear-tipped	p
2014 (from December)	5	0 (0.0%)	-	-	
2015	73	11 (15.1%)	-	-	
2016	48	10 (20.8%)	-	-	
2017	70	9 (13.9%)	54*	4 (7.4%)	0.49
2018 (until January)	12	1 (8.3%)	-	-	
2018 (from February) **	45	6 (13.3%)	316	65 (20.5%)	0.35
**Total (cats used in this study)**	**208**	**31(14.9%)**	**54**	**4 (7.4%)**	
**Total**	**253**	**37 (14.6%)**	**370**	**69 (18.6%)**	

Table S2. Result of factor analysis on land use variables. Factor 1 loaded on the residential area coverage and building density, and factor 2 loaded on farmland coverage and forest coverage.

land use	buffer radius(m)	Factor1	Factor2
farmland coverage	100	-0.292	0.980
	200	-0.208	1.016
	500	-0.035	0.842
forest coverage	100	-0.112	-0.909
	200	-0.184	-0.914
	500	-0.303	-0.761
residential area coverage	100	0.820	0.116
	200	0.875	0.072
	500	0.891	0.030
building density	100	1.015	-0.086
	200	1.021	-0.096
	500	0.920	-0.098
Proportion Var		0.452	0.416

Fig S1. Stable isotope composition of hair from sheltered cats versus time in days. Data were fitted with negative exponential equations. The trophic enrichment factors (TEF) of δ13C and δ15N were defined as 2.3 ± 0.3 and 2.8 ± 0.1, respectively, from the estimated asymptotes.

(a) (b)

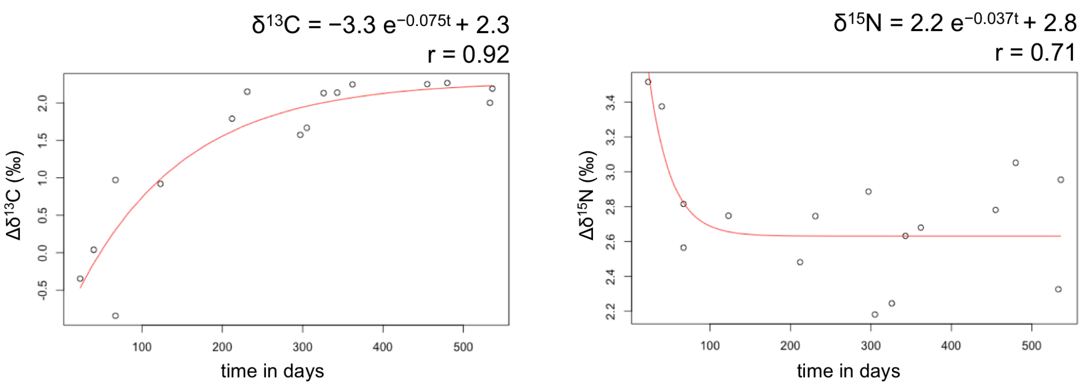

Fig S2. Comparison of the landscape variables between the cats whose feces contained forest-living animals (detected; n = 33) and those that did not (not detected; n = 147). (a) Factor 1 and (b) factor 2 are the land use variables obtained from the factor analysis. Detected individuals had lower value for both factors, i.e., they were caught near the residential area (factor 1) and farther from the forest (factor 2). The error-bars and bold bars in the boxes represent the standard deviations and the medians, respectively.

(a) (b)

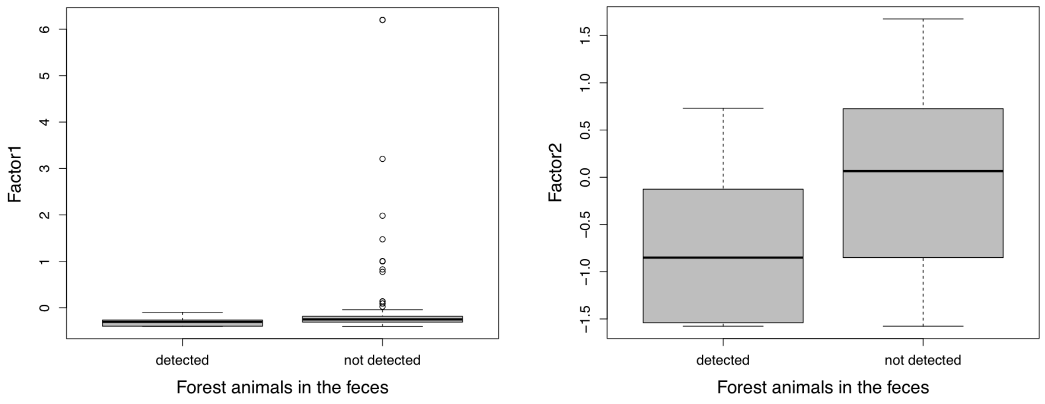

Fig S3. Stable isotope ratio of the cats whose feces contained forest-living animals (detected; n = 33) and those that did not (not detected; n = 147). Both had a high dependency on artificial resources. The error bars represent the 95% highest density regions.

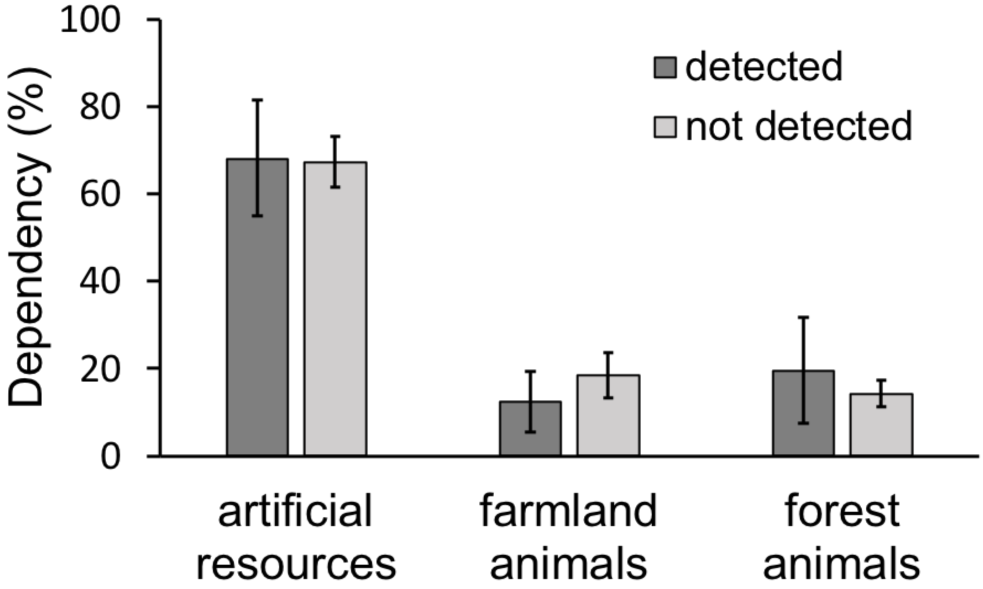

Supplement: Supplementary file 1 — Supplementary Appendix [file 41598_2019_52472_MOESM1_ESM.pdf]
